# Forecasting total and cause-specific health expenditures for 116 health conditions in Norway, 2022–2050

**DOI:** 10.1186/s12916-025-03917-2

**Published:** 2025-02-25

**Authors:** Jonas Minet Kinge, Henning Øien, Joseph L. Dieleman, Bjørn-Atle Reme, Ann Kristin Skrindo Knudsen, Geir Godager, Geir Selbæk, Jan C. Frich, Enis Barış, Christopher J. L. Murray, Stein Emil Vollset

**Affiliations:** 1 Department of Health Management and Health Economics, Faculty of Medicine, University of Oslo, Oslo, Norway; 2 Norwegian Institute of Public Health, Postboks 222 Skøyen, Oslo, 0213 Norway; 3 Department of Health Metrics Sciences and Institute for Health Metrics and Evaluation, University of Washington, Seattle, WA USA; 4 Centre for Disease Burden, Norwegian Institute of Public Health, Oslo, Norway; 5 The Norwegian National Centre for Ageing and Health, Vestfold Hospital Trust, Tønsberg, Norway; 6 Institute of Clinical Medicine, University of Oslo, Oslo, Norway; 7 Department of Geriatric Medicine, Oslo University Hospital, Oslo, Norway

**Keywords:** Health expenditures, Forecasting, Health policy, Behavioral risk factors, Aging

## Abstract

**Background:**

This study forecasts total and cause-specific health expenditures in Norway to 2050 and quantifies the contribution of four key drivers—total population growth, population aging, changes in disease prevalence, and cost per case—on future health care spending.

**Methods:**

We forecast spending for 116 health conditions in Norway from 2022 to 2050, using historical and forecasted data of population growth, disease prevalence, gross domestic product (GDP), health spending, and residual factors. Our analysis included a reference scenario that forecasted disease-specific health spending; two alternative scenarios examining the effects of alternative unit cost developments; and a scenario examining the consequences of improved behavioral and metabolic risk factors.

**Results:**

Health spending increased from 10.6% (95% uncertainty interval, 10.2–11.1) of GDP in 2022 to 14.3% (13.0–15.7) in 2050 in the reference scenario. Among the top aggregate causes of Norwegian health spending in 2022, the spending for neurological disorders rose the most, from 1.7% (1.6–1.8) to 2.7% (2.3–3.1) of GDP, surpassing mental and substance use disorders which rose from 2.2% (2.1–2.3) to 2.4% (2.2–2.6) of GDP. Of the 116 single conditions analyzed, dementias accounted for the highest spending in 2022. This expenditure was forecasted to increase considerably from 1.1% (1.09–1.2) to 1.9% (1.6–2.2) of GDP by 2050, largely due to population aging. Spending on other old-age-related conditions like falls, stroke, and diabetes, was also forecasted to increase. Increased population, aging, and spending per case contributed to increased future spending. Reduced behavioral and metabolic risks were forecasted to increase the number of elderly persons and reduce age-specific disease prevalence but had little impact on forecasted health spending.

**Conclusions:**

Health spending growth was forecasted regardless of the scenario, and Norway needs to plan for this. However, policymakers can curb total spending growth, while maintaining health care quality and output, by ensuring more efficient allocation and effective use of resources. While the overall impact of behavioral and metabolic risk reductions on total healthcare spending was modest, reducing risk factors is needed if countries aim to achieve a healthier, longer-living population.

**Supplementary Information:**

The online version contains supplementary material available at 10.1186/s12916-025-03917-2.

## Background

In most developed countries the healthcare systems are under increasing pressure due to an aging population, rising expectations for healthcare quality, and costly improvements by technology [1–4]. Norway is no exception [5]. While many measures have been proposed to face these pressures, there is a lack of evidence regarding which levers are most effective in improving system performance. Forecasts of health expenditures can provide estimates of the economic consequences of such measures. Traditional models for forecasting health expenditures are typically based on demographic forecasts, macroeconomic indicators on economic development, and measures of technological progress [2–4]. However, there is a scarcity of models capturing developments in disease-specific expenditures, with Australia as a notable exception [6]. This study utilizes detailed administrative register data from Norway to forecast disease-specific expenditures by introducing a model that integrates new epidemiological forecasts with disease-specific spending studies, forecasted gross domestic product (GDP), and technological development. Doing so enables the estimation of future health spending for 116 health conditions.

Norway consistently ranks top or near the top in health system performance among high-income countries [1, 7]. The Norwegian population is healthy, with a 2021 life expectancy of 83.3 years and a healthy life expectancy (HALE) at birth of 71.4 years. In comparison, the average life expectancy and HALE in countries that are part of the Organisation for Economic Co-operation and Development (OECD) are 79.2 and 67.7 years, respectively [1, 8]. While Norway do not face fiscal space constraints to the extent that many other developed countries do, there is room for improving efficiency in the way its health budget is allocated across different cost categories and thus improve its sectoral performance in the longer term [5]. Several measures have been discussed, including more efficient use of personnel, geographical consolidation, and the implementation of new cost-effective technologies [5, 9, 10]. Other strategies, such as reductions in health risk factors and managing patient expectations, have also been mentioned [5]. Yet, the relative implications of these measures largely remain unknown.

The objectives of the present study were to (1) forecast Norway’s health expenditures by diseases and injuries from 2022 to 2050 by integrating disease prevalence with demographic and non-demographic factors for 116 diseases and injuries, (2) to explore alternative scenarios for how healthcare spending is affected by changes in GDP per capita and residual spending growth, (3) to explore how healthcare spending is affected by a gradual elimination of a selection of important risk factors, and (4) to decompose future healthcare spending by its main drivers, for each health condition.

## Methods

This paper estimates health spending from 2022 to 2050 for 116 health conditions identified and categorized by the Norwegian Health Spending Project and the Institute for Health Metrics and Evaluations [11, 12]. All the data sources used in this study and references are listed in Additional file 1: Supplemental Table 1 [11, 13–21].

In our framework, the drivers of disease-specific health spending can be divided into three categories. The first category represents the population’s need for health services by population size, age distribution, and the prevalence of health conditions. The second driver accounts for how changes in GDP per capita affect both the demand for healthcare and the supply of key services (e.g., increased wages for healthcare workers). The final driver represents excess residual growth, which is the growth in health spending, adjusted for need variables and GDP per capita growth. This residual growth reflects how relative prices, frequency of treatment (partially because of increased demand for health care), and technological progress increase health spending. Estimates by C. De la Maisonneuve and JO Martins [22] also suggest that factors like policy changes and institutional shifts are partially captured by including residual growth [22–24].

The forecasting process can be divided into four steps. First, we estimated the association between health spending per case with GDP per capita growth and a residual growth. Second, we used forecasted GDP per capita and residual growth to estimate future disease-specific costs per case. Third, we forecasted health spending from 2022 to 2050 for each age and health condition combination based on forecasted spending per case and forecasted prevalence. Fourth, we aggregated spending for each health condition and across conditions.

Step 1: To estimate the association of GDP per capita and residual growth with health spending per case, we used national data primarily from OECD Health expenditure and financing [13] and the Institute for Health Metrics and Evaluation (IHME) on prevalence by disease [16] for the years 1990 to 2019 (Additional file 1: Supplemental Table 1). Following K. Dybczak and B. Przywara [23] and C. De la Maisonneuve and JO Martins [22] we included data from several countries (i.e., Norway, Sweden, and Denmark) in this part of the analysis to increase precision and make the model less vulnerable to random noise. We estimate the following models [3, 23, 25]:

1 $${\text{lnHCE}}_{c,t}=\alpha^{\text{HCE}}+\beta_1^{\text{HCE}}{\textrm{lnGPD}}_{c,t}+\beta_2^{\text{HCE}}{\textrm{trend}}_t+X_{c,t}'\gamma^{\text{HCE}}+\gamma_c^{\text{HCE}}+\varepsilon_{c,t}^{\text{HCE}},$$

2 $${\text{lnLTC}}_{c,t}=\alpha^{\text{LTC}}+\beta_1^{\text{LTC}}{\textrm{lnGPD}}_{c,t}+\beta_2^{\text{LTC}}{\textrm{trend}}_t+X_{c,t}'\gamma^{\text{LTC}}+\gamma_c^{\text{LTC}}+\varepsilon_{c,t}^{\text{LTC}},$$

where lnHCE and lnLTC are respectively the logarithms of curative health care (general practitioners; other curative outpatient care (like physiotherapists and chiropractors); specialized outpatient curative care; day patient; inpatient and prescription drugs) and long-term care (home-based care; and nursing homes) spending per prevalent case in country c, at time t. Income elasticity by lnGDP per capita was measured by *β*_1_. The impact of residual growth, which primarily reflects technological advancement, on health expenditure was estimated by the *β*_2_’s, which are linear trends that vary by type of care [23, 26]. Given that the forecasting model, in steps 2–4, relied on the number of prevalent cases, the term ${X}_{c,t}{\prime}$ represents a vector of control variables, chosen to adjust for changing population patterns in the causes of disease and their severity. These control variables included: the proportion of the health conditions in population (non-communicable diseases, infectious diseases, and proportion with injuries [omitted]), deaths per prevalent case, and prevalent cases per person. The primary estimation method was a Mixed Linear Model fitted using reduced maximum likelihood, allowing for country random intercepts (${\gamma }_{c})$ (see Additional file 1, Part 1 and Part 2, for a more detailed explanation of this model) [3, 23, 25–34].

Step 2: Age/health condition/type of care-specific per prevalent case spending profiles were estimated for 2022:

3 $${\overline c}_{d,a,i,2022}=\frac{{\text{Expenditures}}_{d,a,i,2022}}{{\textrm{PrevalentCases}}_{d,a,2022}}$$

where ${\overline{c} }_{d,a,i,t}$ is the spending per case of health condition *d*, in age group *a*, for type of care *i*, at time *t.* Data on disease-specific expenditures for Norway was from Kinge et al. [11] and data on cases were from the GBD 2021 Forecasting Collaborators’ study [20] (Additional file 1: Supplemental Table 1). The spending per case was assumed to grow over time with the income elasticity (${\beta }_{1,}$) and residual growth (${\beta }_{2}$) from Eq. 1 and Eq. 2, and spending per prevalent case in a forecasted year *t* was:

4 $${\overline c}_{d,a,i,t}={\overline c}_{d,a,i,t-1}\ast\left(1+\left(\frac{{\text{GDP}}_t-{\text{GDP}}_{t-1}}{{\textrm{GDP}}_{t-1}}\ast\beta_{1,i}\right)+\beta_{2,i}\right),t>2022$$

Forecasted growth in GDP per capita, was from the IHME version Y2023M01D13 and is also shown in the Additional file 1: Supplemental Fig. 1 [15].

A long-run growth rate in healthcare spending that exceeds GDP growth is unsustainable, both from a technical and theoretical point of view. From a technical perspective, a higher healthcare spending growth rate over time would result in healthcare consuming an unrealistically high proportion of national income, approaching the full national budget in the limit [24]. Theoretically, there are diminishing returns to investment in healthcare. Hence, at some point, other domains of governmental spending would be more welfare enhancing, leading to a tapering off of healthcare spending growth. To address this, comparable models assume that impacts of GDP per capita and technology on spending converge to some share of GDP per capita in the long run [23, 30, 33]. Convergence rules for income elasticity and residual growth were thus applied in Eq. 4, where income elasticity reflected ${\beta }_{1}$ in the base-year, converging to unity by 2050, while ${\beta }_{2}$ converged to zero by 2050 [22, 23].

Step 3: Health spending was then forecasted for each age and health condition combination:

5 $${\text{Spending}}_{a,d,i,t}={\text{TotalPop}}_t\ast\frac{{\text{AgeGroupPop}}_{a,t}}{{\textrm{TotalPop}}_t}\ast\frac{{\text{PrevalentCasesAgeGroup}}_{a,d,t}}{{\textrm{AgeGroupPop}}_{a,t}}\ast{\overline c}_{a,d,i,t^{,}}$$

where demographic and epidemiological data was from the GBD 2021 Forecasting Collaborators’ study [20].

This project considered three health conditions not included in the GBD 2021 Forecasting Collaborators’ study [20]: well care and pregnancy-related care; impairments; and, the treatment of risk factors. Well care and pregnancy-related care included general medical examinations, pregnancy and postpartum care, family planning, donor, other counseling services, and social services. Impairment contains care for heart failure, septicemia, and renal failure. The treatment of risk factors, contained tobacco cessation interventions, treatment of obesity, treatment of hypertension and treatment of hyperlipidemia (see Additional file 1, Part 3 and Supplemental Table 2 for more details about the 116 health conditions) [11, 12]. These health conditions were modeled by varying the total population, aging, and spending per case while excluding prevalent cases.

Step 4: The expenditures were then summed over age groups *a*, to estimate the total health spending for health condition *d* in year *t*.

6 $${\text{Spending}}_{d,i,t}=\sum\nolimits_{a=1}^{19}{\text{Spending}}_{a,d,i,t}$$

The expenditures were then summed across all health conditions *d*, to estimate the health spending by type of care—curative health care (HCE) and long-term care (LTC)—for year *t*.

7 $${\text{Spending}}_{i,t}=\sum\nolimits_{d=1}^{116}{\text{Spending}}_{d,i,t}$$

HCE was also summed across years to calculate total health spending (THE). In addition to the reference scenario, a scenario for epidemiological growth, cost pressures and improved behavioral and metabolic risk factors were produced (see Table 1 for a description).

**Table 1:** Scenarios

Scenario	Interpretation	Operation
Reference scenario	This is the baseline scenario	Used forecasted population, age distribution, and prevalence from the reference scenario from GBD 2021 Forecasting Collaborators. The income elasticities and residual growth were initially set to values found in Additional file 1: Supplemental Table 3. Convergence rules were applied in which income elasticity converges to 1 and residual growth to 0 by 2050
Epidemiological growth scenario	This scenario is based on the reference scenario but assumes no residual growth and “neutral” cost per case development	Same as the reference scenario, but the residual growth is set to 0 and income elasticity is set to 1. This means that health spending per case evolves in line with GDP. If no change in the prevalence of diseases occurs, the proportion of spending to GDP will be constant
Cost pressures scenario	This scenario is based on the reference scenario but assumes increased residual growth in spending due to technology and price increases	Same as the reference scenario, but no convergence rules are applied
Improved behavioral and metabolic risk factors scenario	This scenario is based on the reference scenario but assumes a linear elimination by 2050 of health conditions caused by: high BMI, non-optimal diet, smoking, high systolic blood pressure, high LDL cholesterol, and high fasting plasma glucose	Used forecasted population, age distribution, and prevalence from the improved behavioral risk scenario from GBD 2021 Forecasting Collaborators. The income elasticities and residual growth were identical to those in the reference scenario

To estimate how the forecasts were associated with population growth, aging, disease prevalence, and spending, we decomposed the forecasted total and cause-specific health expenditures into additive components of change and assessed their relative importance using the Das Gupta decomposition [35, 36].

To characterize the uncertainty of the estimated coefficients and input parameters, probabilistic sensitivity analysis with 1000 draws from Gaussian distributions was used, based on means and standard errors from the input data [37]. For the prevalence data, Poisson distributions were used. The residual growth, GDP, and the income elasticity were assumed constant across all age, health condition, and year combinations. In contrast, prevalence was drawn independently for each health condition. The reported uncertainty intervals (UIs) were the means and 2.5th and 97.5th percentiles of the 1000 estimates.

To evaluate the performance of the forecasting models we withheld data after 2009 and forecasted health spending. We then compared predicted values with actual values from national health accounts for the years 2010 to 2019. We also calculated the root mean squared error (RMSE), mean absolute error (MAE), mean absolute percentage error (MAPE), and the *R*^2^. Following CD Lewis [38] a MAPE lower than 5% was considered highly accurate, 10–20% good, 20–50% reasonable, and > 50% inaccurate forecasting.

All values were in 2019 Billion NOK (BNOK) based on the GDP deflator from OECD [17, 39]. All analyses were conducted in StataSE 18.0.

## Results

The income elasticities indicated that for each 1% increase in GDP per capita, HCE and LTC spending per case rose by 0.554% (S.E. 0.087) and 1.770% (S.E. 0.183), respectively. The semi-elasticities for residual growth rates showed annual growth rates in HCE and LTC spending per case -independent of GDP per capita growth- of 1.3% (S.E. 0.3) and 0.77% (S.E. 0.5), respectively. The estimates varied by specification (Additional file 1: Supplemental Table 3). The MAPEs were 4.4%, 4.2%, and 6.6% in the reference, cost pressures, and epidemiological growth scenarios, respectively (Additional file 1: Supplemental Table 4 and Supplemental Figs. 2 and 3).

**Fig. 1:**
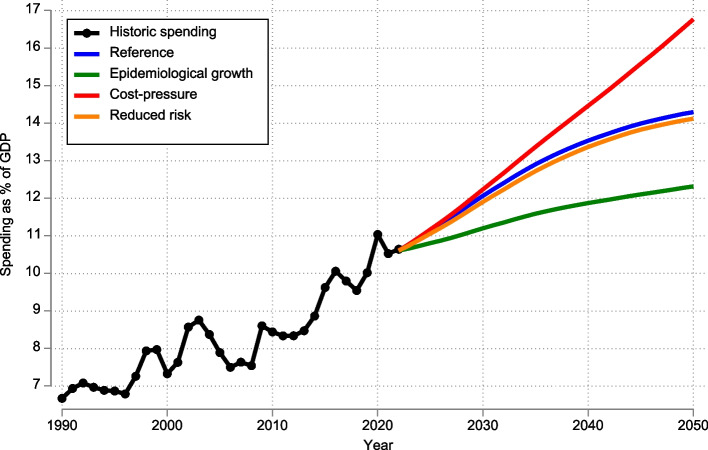
Historic and four forecasted scenarios for health spending as % of GDP*. Notes: *Historic health account values were from OECD Health expenditure and financing [13] and Statistics Norway [19]. Figures for the two last years are preliminary. Historic GDP values were from OECD Economic Outlook 109, and the years 2021 and 2022 are forecasts [21]

**Fig. 2:**
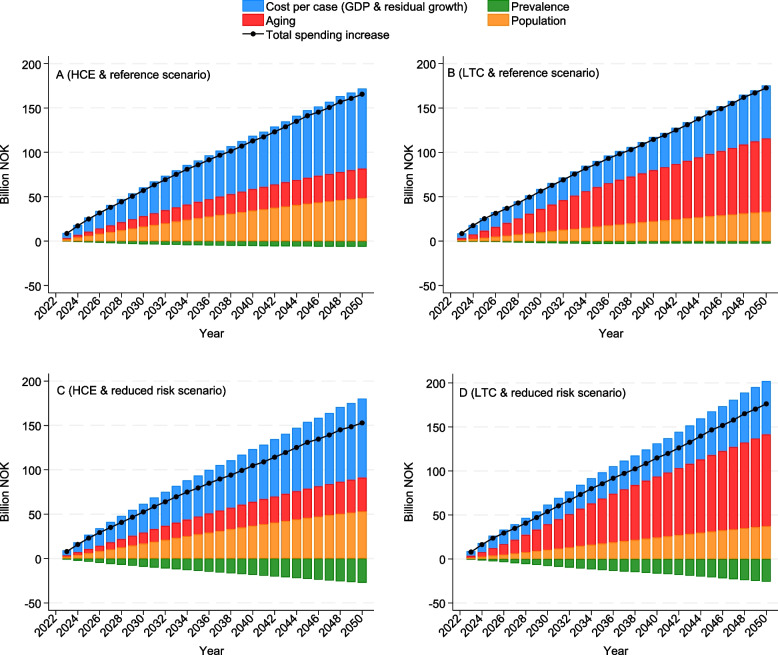
Decomposed health spending changes associated with unit costs, disease prevalence, aging, and total population, reference scenario from 2023 to 2050. For health care expenditures and long-term care expenditures in the reference scenario (**A** and **B**) and in the reduced risk scenario (**C** and **D**)

In the reference scenario, the total health spending was forecasted to increase from 2022 to 2050, from 10.6% of GDP (95% UI 10.2–11.1) in 2022 to 14.3% (13.0–15.7) in 2050 (Fig. 1). Both HCE and LTC contributed to this growth (Additional file 1: Supplemental Fig. 4). In absolute BNOK, the increase in spending on LTC of 173 BNOK was more pronounced than for spending on HCE of 166 BNOK (Fig. 2). We observe that 26.3% of the increase in LTC was due to increased cost per case, while this constituted 66.3% of the increase for HCE. Conversely, 63.2% of the increase in spending for LTC was due to aging, which was much higher than for HCE of 16.7% (Fig. 2).

Both GDP and residual growth contributed to forecasted growth in spending. Residual growth made a larger contribution to total and curative health spending, whereas GDP accounted for a greater share of LTC spending (Additional file 1: Supplemental Fig. 5).

**Fig. 3:**
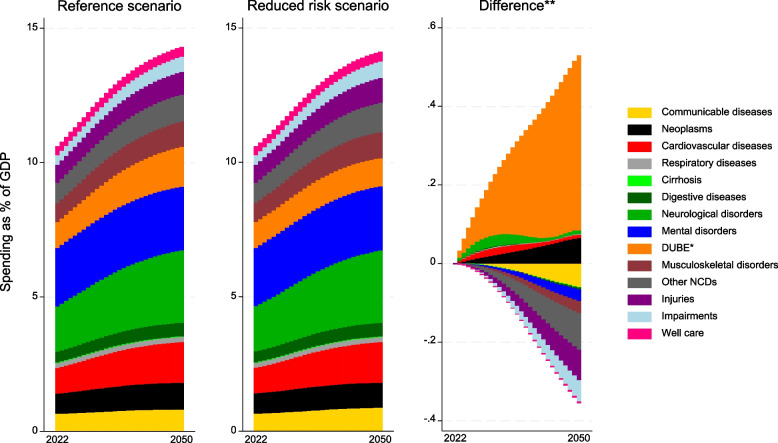
Health spending by aggregated causes, reference scenario, and reduced risk scenario, 2022–2050. *DUBE indicates diabetes, urogenital, blood, and endocrine diseases. **The difference is calculated by subtracting spending in the reduced risk scenario, from spending in the reference scenario

Among the 14 aggregate health conditions, neurological disorders increased the most from 1.7% (1.6–1.8) to 2.7% (2.3–3.1) of GDP, surpassing mental and substance use disorders, which increased from 2.2% (2.1–2.3) to 2.4% (2.2–2.6) of GDP by 2037 (Fig. 3). Rising spending was also forecasted for most of the other aggregate causes, including cardiovascular diseases; diabetes, urogenital, blood, and endocrine diseases; and neoplasms.

Among the 116 health conditions, the highest spending was estimated for dementia in 2022 and in 2050 of 42.61 BNOK (41.52–43.70) and 98.78 (85.57–113.92), respectively (Table 2). Dementia also accounted for the largest increase in spending from 2022–2050, both in absolute BNOK and as a percent of GDP. Most of this increase in spending for dementia from 2022 to 2050, was due to aging alone (Table 2). Large increases in spending were also seen for cerebrovascular disease, unintentional injuries (incl. falls), diabetes mellitus, and sense organ diseases, of which all had a large increase in spending due to aging. Mental disorders, with the highest spending in 2022, like idiopathic intellectual disability, schizophrenia, anxiety disorders, depressive disorders, and drug use disorders, were all more prevalent in younger ages and thus associated with low increases in spending (Table 2).

**Table 2:** Total and per thousand GDP health spending for the 100 most expensive health conditions, and contribution of four factors to changes in health spending from 2022 and 2050

						**Contribution of four factors to changes in spending from 2022 and 2050 (Billion NOK)**			
Rank 2050	Cause name	2022 Billion NOK	2050 Billion NOK	2022 per GDP/1000	2050 per GDP/1000	Pop	Aging	Prev	Unit cost
1	Dementias	42.61 (41.52 to 43.70)	98.78 (85.57 to 113.92)	11.18 (10.90 to 11.47)	19.02 (16.5 to 21.94)	9.89	32.76	− 4.38	17.89
2	Stroke	19.40 (18.91 to 19.89)	44.29 (39.30 to 49.84)	5.09 (4.96 to 5.22)	8.53 (7.57 to 9.60)	4.44	12.37	0.02	8.06
3	Unintentional injuries	22.70 (22.54 to 22.87)	39.45 (37.15 to 41.76)	5.96 (5.91 to 6.00)	7.60 (7.15 to 8.04)	4.43	8.08	− 3.90	8.14
4	Diabetes mellitus	11.79 (11.63 to 11.94)	29.00 (26.66 to 31.72)	3.09 (3.05 to 3.13)	5.58 (5.13 to 6.11)	2.81	5.64	3.65	5.11
5	Sense organ diseases	12.38 (12.31 to 12.45)	25.63 (23.96 to 27.45)	3.25 (3.23 to 3.27)	4.94 (4.61 to 5.29)	2.67	5.84	− 0.15	4.89
6	Intellectual disability	18.70 (17.12 to 20.35)	25.26 (21.26 to 29.82)	4.91 (4.49 to 5.34)	4.86 (4.09 to 5.74)	3.19	− 1.12	− 1.32	5.81
7	Schizophrenia	12.97 (12.18 to 13.77)	19.68 (17.92 to 21.51)	3.40 (3.20 to 3.61)	3.79 (3.45 to 4.14)	2.35	− 0.07	0.12	4.31
8	Skin	11.26 (11.19 to 11.33)	18.89 (17.61 to 20.28)	2.95 (2.94 to 2.97)	3.64 (3.39 to 3.91)	2.16	3.59	− 2.08	3.96
9	Impairments	7.86 (7.83 to 7.89)	18.35 (17.13 to 19.72)	2.06 (2.05 to 2.07)	3.53 (3.30 to 3.80)	1.82	4.84	0.00	3.83
10	WellPreg	12.32 (12.28 to 12.37)	18.34 (17.48 to 19.18)	3.23 (3.22 to 3.25)	3.53 (3.37 to 3.69)	2.22	− 0.80	0.00	4.61
11	Urinary dis. and male infert	8.64 (8.51 to 8.76)	18.30 (17.34 to 19.22)	2.27 (2.23 to 2.30)	3.52 (3.34 to 3.70)	1.88	3.53	0.81	3.44
12	Anxiety disorders	12.16 (11.99 to 12.32)	17.88 (17.10 to 18.71)	3.19 (3.15 to 3.23)	3.44 (3.29 to 3.60)	2.17	− 0.48	0.03	4.00
13	Depressive disorders	10.91 (10.73 to 11.08)	17.61 (16.71 to 18.50)	2.86 (2.82 to 2.91)	3.39 (3.22 to 3.56)	2.04	0.98	− 0.09	3.76
14	Other MSK	9.55 (9.39 to 9.71)	17.43 (16.68 to 18.19)	2.51 (2.46 to 2.55)	3.36 (3.21 to 3.50)	1.91	0.80	1.64	3.52
15	Lower resp. tract infect	8.27 (6.47 to 10.07)	14.93 (11.57 to 18.21)	2.17 (1.70 to 2.64)	2.87 (2.23 to 3.51)	1.65	3.30	− 1.31	3.02
16	Low back and neck pain	8.56 (8.48 to 8.64)	14.11 (13.48 to 14.71)	2.25 (2.23 to 2.27)	2.72 (2.60 to 2.83)	1.62	1.06	− 0.13	2.99
17	Other digestive diseases	7.04 (6.94 to 7.14)	12.62 (11.87 to 13.42)	1.85 (1.82 to 1.87)	2.43 (2.29 to 2.58)	1.40	2.11	− 0.49	2.56
18	Parkinson's disease	5.51 (5.15 to 5.90)	12.49 (10.83 to 14.40)	1.45 (1.35 to 1.55)	2.40 (2.09 to 2.77)	1.25	3.10	0.36	2.27
19	Epilepsy	5.73 (5.44 to 6.06)	12.31 (10.88 to 13.99)	1.50 (1.43 to 1.59)	2.37 (2.10 to 2.69)	1.25	1.44	1.63	2.27
20	Osteoarthritis	6.35 (6.28 to 6.41)	12.06 (11.48 to 12.58)	1.67 (1.65 to 1.68)	2.32 (2.21 to 2.42)	1.30	1.99	0.02	2.40
21	Endoc./metab./blood./immune	5.83 (5.75 to 5.90)	11.49 (10.76 to 12.27)	1.53 (1.51 to 1.55)	2.21 (2.07 to 2.36)	1.21	1.74	0.49	2.22
22	Drug use disorders	6.92 (6.69 to 7.15)	11.23 (10.62 to 11.80)	1.82 (1.75 to 1.88)	2.16 (2.04 to 2.27)	1.30	− 0.47	1.08	2.40
23	Ischemic heart disease	5.88 (5.77 to 6.00)	11.15 (10.65 to 11.67)	1.54 (1.51 to 1.57)	2.15 (2.05 to 2.25)	1.20	1.75	0.10	2.22
24	RiskFactors	5.76 (5.74 to 5.78)	10.94 (10.42 to 11.42)	1.51 (1.51 to 1.52)	2.11 (2.01 to 2.20)	1.18	1.54	0.00	2.46
25	Atrial fibrillation and flutter	4.74 (4.62 to 4.87)	10.91 (10.33 to 11.45)	1.24 (1.21 to 1.28)	2.10 (1.99 to 2.21)	1.08	2.61	0.49	1.99
26	Chronic kidney diseases	4.51 (4.46 to 4.56)	9.88 (9.41 to 10.31)	1.18 (1.17 to 1.20)	1.90 (1.81 to 1.99)	1.00	1.69	0.85	1.84
27	Gynecological	5.74 (5.70 to 5.79)	8.43 (7.90 to 9.00)	1.51 (1.50 to 1.52)	1.62 (1.52 to 1.73)	1.04	1.51	− 1.78	1.91
28	Other CVD	3.98 (3.92 to 4.03)	8.14 (7.76 to 8.51)	1.04 (1.03 to 1.06)	1.57 (1.49 to 1.64)	0.85	1.75	0.00	1.56
29	Alcohol use	5.07 (4.92 to 5.22)	8.08 (7.50 to 8.70)	1.33 (1.29 to 1.37)	1.56 (1.44 to 1.67)	0.94	0.34	0.00	1.73
30	Colon and rectum canc	4.15 (3.96 to 4.34)	7.38 (6.93 to 7.83)	1.09 (1.04 to 1.14)	1.42 (1.33 to 1.51)	0.82	1.29	− 0.38	1.51
31	Bipolar disorder	4.67 (4.49 to 4.86)	7.25 (6.82 to 7.68)	1.23 (1.18 to 1.28)	1.40 (1.31 to 1.48)	0.86	0.14	0.00	1.58
32	COPD	4.23 (4.17 to 4.30)	7.23 (6.88 to 7.56)	1.11 (1.09 to 1.13)	1.39 (1.32 to 1.46)	0.82	1.49	− 0.82	1.51
33	Multiple sclerosis	4.66 (4.27 to 5.07)	7.07 (6.28 to 8.01)	1.22 (1.12 to 1.33)	1.36 (1.21 to 1.54)	0.84	0.29	− 0.27	1.54
34	Other neurological	3.94 (2.95 to 4.91)	6.93 (5.24 to 8.90)	1.03 (0.77 to 1.29)	1.33 (1.01 to 1.71)	0.77	1.07	− 0.26	1.41
35	Neonatal disorders	4.87 (4.73 to 5.01)	6.48 (6.12 to 6.81)	1.28 (1.24 to 1.32)	1.25 (1.18 to 1.31)	0.83	− 0.90	0.15	1.53
36	Congenital anomalies	4.21 (4.05 to 4.37)	6.21 (5.71 to 6.79)	1.11 (1.06 to 1.15)	1.20 (1.10 to 1.31)	0.75	− 0.15	0.02	1.38
37	Breast cancer	3.15 (3.01 to 3.29)	5.41 (5.08 to 5.74)	0.83 (0.79 to 0.86)	1.04 (0.98 to 1.11)	0.61	0.63	− 0.11	1.13
38	Prostate cancer	2.21 (2.13 to 2.30)	5.14 (4.84 to 5.45)	0.58 (0.56 to 0.60)	0.99 (0.93 to 1.05)	0.51	1.00	0.49	0.93
39	Other infectious	2.30 (2.09 to 2.50)	5.03 (4.49 to 5.60)	0.60 (0.55 to 0.66)	0.97 (0.86 to 1.08)	0.51	0.93	0.37	0.93
40	ADHD	3.57 (3.42 to 3.73)	4.78 (4.50 to 5.09)	0.94 (0.90 to 0.98)	0.92 (0.87 to 0.98)	0.61	− 0.52	0.01	1.12
41	Other mental	3.14 (3.05 to 3.22)	4.62 (4.39 to 4.84)	0.82 (0.80 to 0.85)	0.89 (0.85 to 0.93)	0.56	− 0.11	0.00	1.03
42	Lung cancers	2.65 (2.37 to 2.93)	4.40 (3.89 to 4.91)	0.70 (0.62 to 0.77)	0.85 (0.75 to 0.95)	0.50	0.70	− 0.39	0.93
43	Rheumatoid arthritis	2.15 (2.05 to 2.24)	4.29 (4.00 to 4.59)	0.56 (0.54 to 0.59)	0.83 (0.77 to 0.88)	0.45	0.64	0.23	0.83
44	Other neoplasms	2.45 (2.39 to 2.52)	4.29 (4.07 to 4.49)	0.64 (0.63 to 0.66)	0.83 (0.78 to 0.86)	0.48	0.45	0.02	0.88
45	Gallbladder and bil	2.20 (2.18 to 2.23)	3.78 (3.61 to 3.95)	0.58 (0.57 to 0.59)	0.73 (0.69 to 0.76)	0.43	0.66	− 0.29	0.78
46	IBD	2.48 (2.32 to 2.63)	3.65 (3.38 to 3.92)	0.65 (0.61 to 0.69)	0.70 (0.65 to 0.75)	0.44	0.06	− 0.15	0.82
47	Upper resp. tract infect	2.38 (2.32 to 2.44)	3.37 (3.19 to 3.54)	0.62 (0.61 to 0.64)	0.65 (0.61 to 0.68)	0.42	− 0.19	0.00	0.77
48	Transport injuries	1.76 (1.72 to 1.81)	2.85 (2.70 to 3.00)	0.46 (0.45 to 0.47)	0.55 (0.52 to 0.58)	0.33	0.22	− 0.07	0.61
49	Multiple myeloma	1.51 (1.27 to 1.76)	2.85 (2.39 to 3.33)	0.40 (0.33 to 0.46)	0.55 (0.46 to 0.64)	0.31	0.38	0.09	0.57
50	Eating disorders	2.17 (2.05 to 2.30)	2.84 (2.65 to 3.04)	0.57 (0.54 to 0.60)	0.55 (0.51 to 0.59)	0.36	− 0.32	− 0.05	0.67
51	Brain cancers	1.57 (1.41 to 1.74)	2.84 (2.49 to 3.19)	0.41 (0.37 to 0.46)	0.55 (0.48 to 0.61)	0.31	0.37	0.00	0.57
52	Anemia	1.27 (1.24 to 1.30)	2.71 (2.57 to 2.85)	0.33 (0.33 to 0.34)	0.52 (0.49 to 0.55)	0.28	0.66	0.00	0.51
53	Non-melan. skin cancer	1.27 (1.05 to 1.51)	2.68 (2.21 to 3.22)	0.33 (0.28 to 0.40)	0.52 (0.43 to 0.62)	0.28	0.45	0.17	0.51
54	Autistic disorders	2.00 (1.92 to 2.08)	2.65 (2.47 to 2.84)	0.53 (0.50 to 0.55)	0.51 (0.48 to 0.55)	0.34	− 0.32	0.01	0.62
55	Peripheral vascular	1.26 (1.24 to 1.28)	2.43 (2.31 to 2.54)	0.33 (0.32 to 0.34)	0.47 (0.44 to 0.49)	0.26	0.61	− 0.18	0.48
56	Headache	1.62 (1.62 to 1.63)	2.42 (2.32 to 2.53)	0.43 (0.42 to 0.43)	0.47 (0.45 to 0.49)	0.29	− 0.03	0.00	0.54
57	Hernia	1.27 (1.15 to 1.40)	2.24 (1.96 to 2.53)	0.33 (0.30 to 0.37)	0.43 (0.38 to 0.49)	0.25	0.55	− 0.28	0.46
58	Leukemia	1.50 (1.29 to 1.72)	2.22 (1.88 to 2.56)	0.39 (0.34 to 0.45)	0.43 (0.36 to 0.49)	0.27	0.23	− 0.28	0.50
59	Diarrheal diseases	0.73 (0.70 to 0.76)	2.04 (1.93 to 2.16)	0.19 (0.18 to 0.20)	0.39 (0.37 to 0.42)	0.19	0.18	0.60	0.34
60	Malnutrition	0.95 (0.93 to 0.98)	2.00 (1.79 to 2.25)	0.25 (0.24 to 0.26)	0.38 (0.35 to 0.43)	0.21	0.46	0.00	0.38
61	Non-Hodgkin lymph	1.12 (1.02 to 1.23)	1.98 (1.79 to 2.20)	0.29 (0.27 to 0.32)	0.38 (0.34 to 0.42)	0.22	0.27	− 0.04	0.41
62	HIV/AIDS	0.69 (0.62 to 0.77)	1.96 (1.75 to 2.18)	0.18 (0.16 to 0.20)	0.38 (0.34 to 0.42)	0.18	0.03	0.73	0.33
63	Paralytic./intestinal	0.87 (0.65 to 1.10)	1.76 (1.32 to 2.22)	0.23 (0.17 to 0.29)	0.34 (0.25 to 0.43)	0.18	0.28	0.09	0.34
64	Bladder cancer	0.84 (0.77 to 0.91)	1.71 (1.55 to 1.89)	0.22 (0.20 to 0.24)	0.33 (0.30 to 0.36)	0.18	0.36	0.00	0.33
65	Pancreatic cancer	0.76 (0.60 to 0.94)	1.59 (1.24 to 1.97)	0.20 (0.16 to 0.25)	0.31 (0.24 to 0.38)	0.16	0.22	0.14	0.30
66	Malignant skin mel	0.84 (0.79 to 0.88)	1.57 (1.46 to 1.68)	0.22 (0.21 to 0.23)	0.30 (0.28 to 0.32)	0.17	0.24	0.01	0.31
67	Self-harm and violence	1.09 (1.07 to 1.12)	1.53 (1.45 to 1.61)	0.29 (0.28 to 0.29)	0.30 (0.28 to 0.31)	0.19	0.05	− 0.15	0.35
68	Kidney cancer	0.77 (0.68 to 0.86)	1.39 (1.22 to 1.55)	0.20 (0.18 to 0.23)	0.27 (0.24 to 0.30)	0.15	0.17	0.02	0.28
69	Asthma	2.14 (2.10 to 2.18)	1.38 (1.32 to 1.45)	0.56 (0.55 to 0.57)	0.27 (0.25 to 0.28)	0.27	0.12	− 1.66	0.51
70	Oral disorders	0.84 (0.83 to 0.84)	1.35 (1.29 to 1.41)	0.22 (0.22 to 0.22)	0.26 (0.25 to 0.27)	0.16	0.07	0.00	0.29
71	Cirrhosis	0.99 (0.98 to 1.00)	1.35 (1.29 to 1.41)	0.26 (0.26 to 0.26)	0.26 (0.25 to 0.27)	0.17	0.10	− 0.23	0.31
72	Pancreatitis	0.66 (0.61 to 0.72)	1.20 (1.09 to 1.31)	0.17 (0.16 to 0.19)	0.23 (0.21 to 0.25)	0.13	0.17	− 0.01	0.24
73	Ovarian cancer	0.64 (0.53 to 0.77)	1.14 (0.92 to 1.36)	0.17 (0.14 to 0.20)	0.22 (0.18 to 0.26)	0.13	0.13	0.01	0.23
74	Lung disease	0.53 (0.48 to 0.58)	1.10 (0.98 to 1.21)	0.14 (0.13 to 0.15)	0.21 (0.19 to 0.23)	0.11	0.21	0.04	0.21
75	Uterine cancer	0.56 (0.51 to 0.62)	0.99 (0.87 to 1.10)	0.15 (0.13 to 0.16)	0.19 (0.17 to 0.21)	0.11	0.09	0.02	0.20
76	Hepatitis	0.65 (0.56 to 0.73)	0.91 (0.78 to 1.05)	0.17 (0.15 to 0.19)	0.18 (0.15 to 0.20)	0.11	− 0.01	− 0.05	0.21
77	Endocarditis	0.37 (0.27 to 0.47)	0.87 (0.64 to 1.11)	0.10 (0.07 to 0.12)	0.17 (0.12 to 0.21)	0.09	0.14	0.11	0.16
78	Gout	0.36 (0.35 to 0.37)	0.77 (0.73 to 0.80)	0.09 (0.09 to 0.10)	0.15 (0.14 to 0.15)	0.08	0.16	0.03	0.14
79	Appendicitis	0.72 (0.41 to 1.06)	0.74 (0.44 to 1.08)	0.19 (0.11 to 0.28)	0.14 (0.08 to 0.21)	0.11	0.02	− 0.31	0.20
80	Mouth cancer	0.39 (0.32 to 0.46)	0.69 (0.56 to 0.82)	0.10 (0.08 to 0.12)	0.13 (0.11 to 0.16)	0.08	0.10	− 0.02	0.14
81	Liver cancer	0.30 (0.21 to 0.39)	0.64 (0.46 to 0.84)	0.08 (0.06 to 0.10)	0.12 (0.09 to 0.16)	0.06	0.07	0.08	0.12
82	Varicella	0.35 (0.30 to 0.39)	0.63 (0.53 to 0.73)	0.09 (0.08 to 0.10)	0.12 (0.10 to 0.14)	0.07	0.19	− 0.10	0.13
83	Maternal disorders	1.43 (1.20 to 1.68)	0.61 (0.51 to 0.72)	0.38 (0.32 to 0.44)	0.12 (0.10 to 0.14)	0.16	− 0.09	− 1.19	0.30
84	Stomach cancer	0.46 (0.35 to 0.56)	0.60 (0.45 to 0.74)	0.12 (0.09 to 0.15)	0.11 (0.09 to 0.14)	0.08	0.11	− 0.19	0.14
85	Esophageal cancer	0.32 (0.22 to 0.42)	0.54 (0.38 to 0.70)	0.08 (0.06 to 0.11)	0.10 (0.07 to 0.14)	0.06	0.07	− 0.02	0.11
86	Cardiomyo. and myocard	0.38 (0.33 to 0.43)	0.49 (0.43 to 0.55)	0.10 (0.09 to 0.11)	0.09 (0.08 to 0.11)	0.06	0.04	− 0.11	0.12
87	Conduct disorder	0.38 (0.36 to 0.40)	0.48 (0.45 to 0.51)	0.10 (0.09 to 0.10)	0.09 (0.09 to 0.10)	0.06	− 0.08	0.00	0.12
88	Otitis media	0.38 (0.36 to 0.40)	0.39 (0.36 to 0.41)	0.10 (0.09 to 0.10)	0.07 (0.07 to 0.08)	0.06	− 0.03	− 0.12	0.11
89	Meningitis	0.40 (0.31 to 0.49)	0.35 (0.27 to 0.43)	0.10 (0.08 to 0.13)	0.07 (0.05 to 0.08)	0.06	0.05	− 0.27	0.10
90	Thyroid cancer	0.19 (0.16 to 0.23)	0.34 (0.28 to 0.40)	0.05 (0.04 to 0.06)	0.06 (0.05 to 0.08)	0.04	0.03	0.00	0.07
91	Anemias	0.22 (0.21 to 0.22)	0.33 (0.32 to 0.35)	0.06 (0.06 to 0.06)	0.06 (0.06 to 0.07)	0.04	0.01	0.00	0.07
92	Cervical cancer	0.26 (0.21 to 0.32)	0.31 (0.25 to 0.38)	0.07 (0.05 to 0.08)	0.06 (0.05 to 0.07)	0.04	0.02	− 0.09	0.08
93	STD (excl. HIV)	0.23 (0.22 to 0.23)	0.31 (0.30 to 0.33)	0.06 (0.06 to 0.06)	0.06 (0.06 to 0.06)	0.04	− 0.01	− 0.01	0.07
94	Rheumatic heart dis	0.17 (0.14 to 0.19)	0.31 (0.26 to 0.37)	0.04 (0.04 to 0.05)	0.06 (0.05 to 0.07)	0.03	0.05	0.00	0.06
95	Other pharynx canc	0.16 (0.12 to 0.21)	0.28 (0.20 to 0.38)	0.04 (0.03 to 0.06)	0.05 (0.04 to 0.07)	0.03	0.02	0.01	0.06
96	Vascular intestinal	0.16 (0.10 to 0.22)	0.28 (0.17 to 0.39)	0.04 (0.03 to 0.06)	0.05 (0.03 to 0.08)	0.03	0.05	− 0.02	0.06
97	Hodgkin lymphoma	0.18 (0.13 to 0.22)	0.27 (0.20 to 0.33)	0.05 (0.04 to 0.06)	0.05 (0.04 to 0.06)	0.03	0.01	− 0.01	0.06
98	Encephalitis	0.16 (0.09 to 0.23)	0.25 (0.15 to 0.36)	0.04 (0.02 to 0.06)	0.05 (0.03 to 0.07)	0.03	0.01	− 0.01	0.05
99	Gallbladder cancer	0.14 (0.09 to 0.19)	0.19 (0.13 to 0.27)	0.04 (0.02 to 0.05)	0.04 (0.03 to 0.05)	0.02	0.03	− 0.05	0.04
100	Larynx cancer	0.13 (0.09 to 0.16)	0.18 (0.13 to 0.23)	0.03 (0.02 to 0.04)	0.03 (0.03 to 0.04)	0.02	0.03	− 0.04	0.04

Historic health account values were from OECD Health expenditure and financing [13] and Statistics Norway [19] Figures for the two last years are preliminary. Historic GDP values were from OECD Economic Outlook 109, and the years 2021 and 2022 are forecasts [43]

### The epidemiological growth, cost pressures and reduced risk scenarios

We forecasted large differences when varying the growth in cost per case. Spending as a percent of GDP increased from 10.6% in 2022 to 12.3% in 2050, in the epidemiological growth scenario and to 16.8% in the cost pressures scenarios (Fig. 1).

**Fig. 4:**
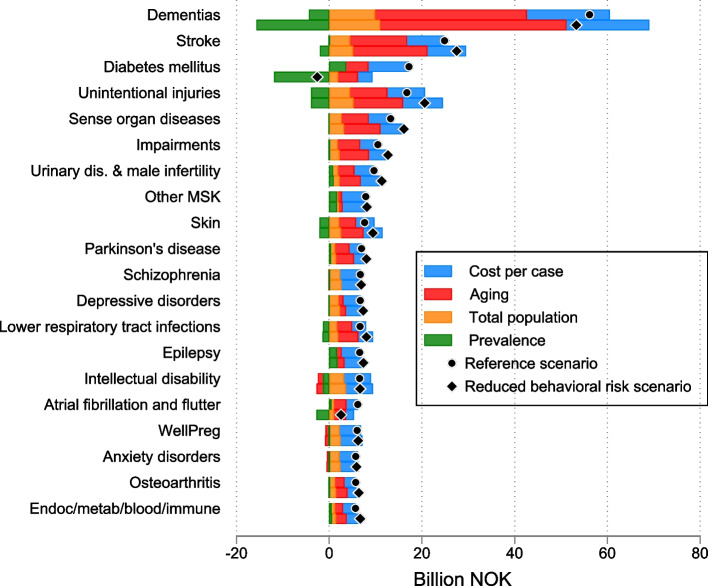
Decomposed changes in health spending associated with unit costs, disease prevalence, aging, and total population for the 20 health conditions with the largest increase in spending in the reference scenario, 2022–2050. Note: Spending on the top 20 health conditions in the figure constitutes 66% of total spending in 2050

Compared with the reference scenario, improved behavioral and metabolic risk factors resulted in lower total spending (Fig. 1). The improvement in behavioral and metabolic risks reduced spending for some conditions and increased spending for others (Figs. 3 and 4). Spending on diabetes in the reference scenario increased from 11.79 BNOK (11.63–11.94) in 2022 to 29.00 BNOK (26.66–31.72) in 2050, while in the reduced behavioral risk scenario, spending on diabetes was reduced to 10.4 BNOK in 2050. However, spending for other conditions, like stroke, unintentional injuries, and sense organ diseases, increased. For dementia, spending increased from 42.6 BNOK (41.5–43.7) in 2022 to 98.78 (85.57–113.92) in 2050 in the reference scenario. While it increased to BNOK 95.9 (83.1–110.7) in 2050 in the reduced behavioral risk scenario (Fig. 4).

Compared with the reference scenario, the forecasts of spending on mental disorders, like schizophrenia, anxiety, and depression, increase in the reduced behavioral risk scenario due to the increased total population (Fig. 3).

## Discussion

This study forecasted spending for health services and long-term care across 116 health conditions in Norway from 2022 to 2050 under four scenarios. While total health spending increased in all scenarios, the distribution by age and health condition varied by year and scenario. The study attributed changes over time to four factors: total population growth, population aging, changes in disease prevalence, and cost per case. Long-term care spending increased more than other services, primarily due to aging. Expenditures for dementia, stroke, injuries, and diabetes were forecasted to rise substantially. The study also highlighted some prospects for reducing future diabetes-related expenditures by reducing behavioral and metabolic risks.

OECD and the European Commission also forecasted total spending for Norway, and our forecasts align with these. The OECD forecasted an increase in health spending from 10.1% of GDP in 2015 to 12.2% in 2030, an average annual growth of 0.14% per year [3]. Similarly, the European Commission estimated that combined health and long-term care spending would rise from 11% of GDP in 2019 to 14.5% by 2050, corresponding to an average growth rate of 0.11% per year [4]. Our forecasts of growth in health spending from 10.6% of GDP in 2022 to 14.3%, and thus an average annual growth of 0.13, is of comparable magnitude.

### Policy implications

Spending on health and long-term care increased in all scenarios, both in absolute terms and as a percentage of GDP, indicates that constant or reduced future spending on health is unlikely. Consequently, the Norwegian government needs to plan for growth in health spending [3].

The forecasts, which were based on the historical relations, suggest that the need for care—proxied by disease prevalence—is likely to account for a portion of the growth in spending, with this portion being higher for LTC. However, other factors, such as GDP per capita growth and residual growth, were forecasted to play a pivotal role in determining the extent of future health spending growth. These other factors could be related to the use of health technology and the organization of services.

To the extent that policy can alter these historical relationships, as reflected by the parameters in this study, spending growth could be mitigated through greater efficiency, while simultaneously retaining and recruiting healthcare personnel.

Given the input from the historical relations and the forecasted prevalence, our forecast suggested that the development in cost-per-case was most important in curative care, with less impact on long-term care. This finding highlights the importance of continued efforts to improve supply-side efficiency, particularly in specialist and primary care. For example, while Norway has the highest number of physicians per capita, it ranks among the lowest in physician consultations per capita [9]. Geographical consolidation might be improved, as specialist health services and elderly care are highly geographically dispersed across a relatively small population. Moreover, utilization of some key and often expensive services, like magnetic resonance imaging (MRI) usage, lies significantly above the OECD average [10]. It will be crucial for governments to harness technological progress effectively, particularly technologies that enhance the efficiency of care provision [3].

The developments on the demand side are largely outside the government’s control. Increased demand due to an aging population was forecasted to substantially increase pressures on long-term care services, particularly for conditions like dementia. However, the upward pressures on health care expenditures from increased demand may be largely mitigated by improving supply-side efficiency while safeguarding population health.

An important aspect of supply-side efficiency, in a wide range of countries including Norway, involves the potential misallocation of resources between sectors, which stems from differing responsibilities: municipalities are responsible for financing primary health care and long-term care, while specialist care is state-funded [40]. Hence, there are potential gains through targeted planning and effective integration, particularly when addressing the future challenges age-related conditions pose for municipal healthcare services [11].

A key demand side factor is the increasing public expectation and willingness to spend on personal health care as GDP grows [4, 15]. Based on the forecasts of increased future GDP, the willingness to spend more on health care services will also grow [2–4]. The demand will likely grow the most among those in most need of care, compared to the supply-side capacity, which will be particularly challenging among the elderly with conditions like dementia [4, 23]. If the government does not accommodate this increased willingness to spend, a shift toward more privately financed services may occur [3].

The impact on healthcare expenditure from changes in behavioral and metabolic risk factors was relatively small. The input data from the GBD 2021 Forecasting Collaborators’ study [20] considered competing risks when forecasting the implications of reductions in risks from behavioral & metabolic risk factors. Compared to the reference scenario, the reduced behavioral and metabolic risk scenario predicted declines in age-specific prevalence for conditions like dementia. At the same time, this scenario also forecasted a larger older population, due to decreased mortality from diseases associated with behavioral risks. Although this shift in spending from younger to older had little impact on total healthcare spending, this scenario will reduce overall disability and premature mortality and thus increase HALE substantially [20]. As such, it will not reduce spending substantially but increase performance and efficiency by having a healthier population living longer. As the population ages, if any related policies could raise the proportion of the population that is working, thereby boosting GDP, it could potentially also mitigate the growth of health care spending as a percentage of GDP.

### Limitations

Long-term forecasting, and especially of health care spending, is inherently uncertain [41]. For example, new technology, like new weight loss treatments, may impact on risk factors and diseases and thereby change the prevalence and management. In addition, this study has several limitations. First, the model only partially accounted for a potential heterogeneous effect of GDP per capita, prices, and technological advancement on the cost per case across different health conditions. It assumed that the development of spending per case was uniform, within the type of care, and proportional to the spending patterns observed in 2019 by age and disease. Second, our estimates may be biased as health spending and GDP could be correlated due to various factors, including unmeasured third variables, that were not accounted for in our regressions [42]. Additionally, the causal relationship between health spending per case and GDP per capita could be bidirectional [43]. Third, the model does not separate treatment proportion, volume of care, intensity of care, and price for each health condition and age group but models these jointly. Fourth, this study relied on estimates and forecasts from other studies, which themselves contain uncertainties due to data limitations, although we did propagate uncertainty intended to capture this. Fifth, the model did not account for any changes in cost per case resulting from future changes in immigration. Also, our estimates assumed constant patterns of spending for comorbidities. However, these comorbidity patterns may change over the coming decades, and our estimates cannot account for such changes. Sixth, while we have tested the model on a “left out” period of historical data, this “left out” period covered only 10 year, which was less than our forecasts of spending of 28 years. Finally, our UIs only captured a subset of uncertainty. They do not capture the uncertainty from the decisions about the model and one of the data sources did not have any estimates of uncertainty. Hence, the UIs should be considered a lower bound.

## Conclusions

Norwegian health spending was forecasted to grow in four scenarios, highlighting the need for policymakers to prepare for this rise. The growth was expected to be more pronounced in long-term care compared to other health services. However, government policies can shape the trajectory of health expenditures, depending on how resources are allocated within the healthcare system.

## Supplementary Information

Additional file 1: Supplementary methods Part 1–3: Part 1. Determinants of health and long-term care expenditures. Part 2. More details about the forecasting methodology. Part 3. The cause list. Supplementary tables 1–4. Supplemental Table 1. Data sources used in this study. Supplemental Table 2. Aggregated to disaggregated reporting level. Supplemental Table 3. Regression results, coefficients and standard errors. Supplemental Table 4. Forecast performance measures. Supplemental Figs. 1–5. Supplemental Fig. 1. Forecasted growth in GDP per capita, with uncertainty intervals. Supplemental Fig. 2. Historic and four forecasted scenarios for health spending, 2009–2019. Supplemental Fig. 3. Historic and four forecasted scenarios for health spending as % of GDP, 2009–2019. Supplemental Fig. 4. Four forecasted scenarios for health spending as % of GDP, for health care expendituresand long-term care expenditures. Supplemental Fig. 5. Four scenarios for spending varying the contribution of GDP per capita growth and residual growth.

## Data Availability

Parts of the data that support the findings of this study are available from helsedata.no but restrictions apply to the availability of these data, which were used under license for the current study, and so are not publicly available. Some of the data is publicly available and described with references in Additional file 1, Supplemental Table 1.

## Abbreviations

BNOK: Billion NOK
GDP: Gross domestic product
HALE: Healthy life expectancy
HCE: Curative health care
IHME: Institute for Health Metrics and Evaluation
LTC: Long-term care
MAE: Mean absolute error
MAPE: Mean absolute percentage error
MRI: Magnetic resonance imaging
OECD: The Organisation for Economic Co-operation and Development
RMSE: Root mean squared error
THE: Total health spending
UI: Uncertainty interval

## References

[1] Schneider EC, Shah A, Doty MM, Tikkanen R, Fields K, Williams R, et al. Reflecting Poorly: Health Care in the US Compared to Other High-Income Countries. New York: The Commonwealth Fund. 2021.

[2] Astolfi R, Lorenzoni L, Oderkirk J. Informing policy makers about future health spending: a comparative analysis of forecasting methods in OECD countries. Health Policy. 2012;107(1):1–10. 22682763 10.1016/j.healthpol.2012.05.001

[3] Lorenzoni L, Marino A, Morgan D, James C. Health spending projections to 2030: New results based on a revised OECD methodology, OECD health working papers. Paris: OECD Publishing; 2019;110.

[4] European Commission. The 2021 Ageing Report. Economic and Budgetary Projections for the EU Member States (2019–2070). European Economy Institutional Paper 148. Luxembourg: Publications Office of the European Union; 2021.

[5] NOU. Tid for Handling — Personellet i En Bærekraftig Helse- Og Omsorgstjeneste. Oslo, Norway: Helse og Omsorgsdepartementet; 2023;4.

[6] Begg S, Vos T, Goss J, Mann N. An alternative approach to projecting health expenditure in Australia. Aust Health Rev. 2008;32(1):148–55. 18241159 10.1071/ah080148

[7] Barber RM, Fullman N, Sorensen RJD, Bollyky T, McKee M, Nolte E, Abajobir AA, Abate KH, Abbafati C, Abbas KM, et al. Healthcare Access and Quality Index based on mortality from causes amenable to personal health care in 195 countries and territories, 1990–2015: a novel analysis from the Global Burden of Disease Study 2015. Lancet. 2017;390(10091):231–66. 28528753 10.1016/S0140-6736(17)30818-8 PMC5528124

[8] Schumacher AE, Kyu HH, Aali A, Abbafati C, Abbas J, Abbasgholizadeh R, Abbasi MA, Abbasian M, Abd ElHafeez S, Abdelmasseh M, et al. Global age-sex-specific mortality, life expectancy, and population estimates in 204 countries and territories and 811 subnational locations, 1950–2021, and the impact of the COVID-19 pandemic: a comprehensive demographic analysis for the Global Burden of Disease Study 2021. Lancet. 2024;403(10440):1989–2056. 38484753 10.1016/S0140-6736(24)00476-8 PMC11126395

[9] Turner A, Miller G, Lowry E. High US Health Care Spending: Where Is It All Going. New York: The Commonwealth Fund; 2023.

[10] Munira Z. Gunja EDG, Reginald D. Williams II: U.S. Health Care from a Global Perspective, 2022: Accelerating Spending, Worsening Outcomes. New York: The Commonwealth Fund; 2023.

[11] Kinge JM, Dieleman JL, Karlstad Ø, Knudsen AK, Klitkou ST, Hay SI, Vos T, Murray CJ, Vollset SE. Disease-specific health spending by age, sex, and type of care in Norway: a national health registry study. BMC Med. 2023;21(1):201. 37277874 10.1186/s12916-023-02896-6 PMC10243068

[12] Dieleman JL, Cao J, Chapin A, Chen C, Li Z, Liu A, Horst C, Kaldjian A, Matyasz T, Scott KW. US health care spending by payer and health condition, 1996–2016. JAMA. 2020;323(9):863–84. 32125402 10.1001/jama.2020.0734 PMC7054840

[13] OECD. OECD Health Statistics—Health Expenditure and Financing. OECD. Paris; 2023. https://www.oecd.org/en/data/datasets/oecd-health-statistics.html.

[14] OECD. Demography and poplation. OECD. Paris: 2023. https://www.oecd.org/en/data/indicators/population.html.

[15] Global Burden of Disease Collaborative Network. Gross Domestic Product Per Capita 1960-2050. Seattle, United States of America: Institute for Health Metrics and Evaluation (IHME); 2021.

[16] Institute for Health Metrics and Evaluation (IHME). GBD Results. Seattle, WA: IHME, University of Washington; 2024. https://vizhub.healthdata.org/gbd-results/.

[17] OECD. National accounts. OECD. Paris: 2023. https://www.oecd.org/en/about/programmes/oecd-data-collection-programme/national-accounts.html.

[18] Statistics Sweden. System of Health Accounts (SHA). Statistics Sweden. Örebro; 2023. https://www.statistikdatabasen.scb.se/pxweb/en/ssd/START__NR__NR0109/HCHF/.

[19] Statistics Norway. Health accounts. Statistics Norway. Oslo; 2023. https://www.ssb.no/en/nasjonalregnskap-og-konjunkturer/nasjonalregnskap/statistikk/helseregnskap.

[20] Vollset SE, Ababneh HS, Abate YH, Abbafati C, Abbasgholizadeh R, Abbasian M, Abbastabar H. Abd Al Magied AH, Abd ElHafeez S, Abdelkader A. Burden of disease scenarios for 204 countries and territories, 2022–2050: a forecasting analsysi for the Global Burden of Disease Study. Lancet. 2024;403(10440):2204–56. 38762325 10.1016/S0140-6736(24)00685-8 PMC11121021

[21] OECD. Economic Outlook 109 database. OECD. Paris: 2021. https://www.oecd.org/en/publications/oecd-economic-outlook/volume-2021/issue-1_490d4832-en.html.

[22] De la Maisonneuve C, Martins JO. The future of health and long-term care spending. OECD Journal: Economic Studies. 2015;2014(1):61–96.

[23] Dybczak K, Przywara B. The role of technology in health care expenditure in the EU. In.: Directorate General Economic and Financial Affairs, European Commission; 2010.

[24] Grignon M. SB, Wang L. Model of Long-Term Health Care Cost Trends in Canada. In.: Canadian Institute of Actuaries and the Society of Actuaries 2018.

[25] Costa-Font J, Vilaplana-Prieto C. ‘Investing’in care for old age? An examination of long-term care expenditure dynamics and its spillovers. Empirical Economics. 2023;64(1):1–30. 35668842 10.1007/s00181-022-02246-0 PMC9137442

[26] Okunade AA, Murthy VN. Technology as a ‘major driver’of health care costs: a cointegration analysis of the Newhouse conjecture. J Health Econ. 2002;21(1):147–59. 11852912 10.1016/s0167-6296(01)00122-9

[27] Gerdtham U-G, Löthgren M. On stationarity and cointegration of international health expenditure and GDP. J Health Econ. 2000;19(4):461–75. 11010235 10.1016/s0167-6296(99)00036-3

[28] Feng Y, Watt T, Charlesworth A, Marsden G, Roberts A, Sussex J. What Determines the Health Care Expenditure of High Income Countries? A Dynamic Estimation. Applied Economics and Finance. 2017.

[29] Thompson Jr W. The problem of negative estimates of variance components. The Annals of Mathematical Statistics. 1962:273–289.

[30] De la Maisonneuve C, Oliveira Martins J. A projection method for public health and long-term care expenditures. OECD Economics Department Working Papers, No. 1048. Paris: OECD Publishing; 2013.

[31] Perry-Duxbury M, Asaria M, Lomas J, van Baal P. Cured today, ill tomorrow: a method for including future unrelated medical costs in economic evaluation in England and Wales. Value in Health. 2020;23(8):1027–33. 32828214 10.1016/j.jval.2020.05.006

[32] Baumol WJ. Health care, education and the cost disease: A looming crisis for public choice. In: The next twenty-five years of public choice. edn.: Dordrecht: Springer Netherlands; 1993. p. 17–28.

[33] Heffler S, Caldis, TG., Smith, SD., Cuckler, GA. The Long-Term Projection Assumptions for Medicare and Aggregate National Health Expenditures. In. Edited by SERVICES DOHAH. Baltimore, MD: Centers for Medicare Medicaid Services.

[34] Wooldridge JM. Introductory Econometrics: A Modern Approach 3rd ed. Mason, Ohio: Thomson South-Western; 2006.

[35] Das Gupta P. Standardization and decomposition of rates: A user's manual, U.S. Bureau of the Census, Current Population Reports, Series P23-186, U.S. Washington, DC: Government Printing Office; 1993.

[36] Dieleman JL, Squires E, Bui AL, Campbell M, Chapin A, Hamavid H, Horst C, Li Z, Matyasz T, Reynolds A. Factors associated with increases in US health care spending, 1996–2013. JAMA. 2017;318(17):1668–78. 29114831 10.1001/jama.2017.15927 PMC5818797

[37] Drummond ME, Sculpher MJ, Torrance GW, O’Brien BJ, Stoddart GL. Methods for the Economic Evaluation of Health Care Programmes. Oxford, United Kingdom: Oxford Univesity press; 2005.

[38] Lewis CD. Industrial and business forecasting methods: A practical guide to exponential smoothing and curve fitting. London: Butterworths; 1982.

[39] OECD. Long-term baseline projections, No. 114. In. Edited by OECD. Paris; 2023.

[40] Saltman R, Busse R, Figueras J. Decentralization in health care: strategies and outcomes: McGraw-Hill Education (UK), Berkshire, England; 2006.

[41] Lee R, Miller T. An approach to forecasting health expenditures, with application to the US Medicare system. Health Serv Res. 2002;37(5):1365–86. 12479501 10.1111/1475-6773.01112 PMC1464029

[42] Mino K, Sasaki H. Long-run consequences of population decline in an economy with exhaustible resources. Econ Model. 2023;121:106212.

[43] Devlin N, Hansen P. Health care spending and economic output: Granger causality. Appl Econ Lett. 2001;8(8):561–4.

