# Supplementary material for: Forecasting total and cause-specific health expenditures for 116 health conditions in Norway, 2022–2050
Source: BMC Med. 2025 Feb 25;23:116. doi: 10.1186/s12916-025-03917-2 (PMC11863442; doi:10.1186/s12916-025-03917-2)
Supplement: Supplementary file 1 — Additional file 1: Supplementary methods Part 1–3: Part 1. Determinants of health and long-term care expenditures. Part 2. More details about the forecasting methodology. Part 3. The cause list. Supplementary tables 1–4. Supplemental Table 1. Data sources used in this study. Supplemental Table 2. Aggregated to disaggregated reporting level. Supplemental Table 3. Regression results, coefficients and standard errors. Supplemental Table 4. Forecast performance measures. Supplemental Figs. 1–5. Supplemental Fig. 1. Forecasted growth in GDP per capita, with uncertainty intervals. Supplemental Fig. 2. Historic and four forecasted scenarios for health spending, 2009–2019. Supplemental Fig. 3. Historic and four forecasted scenarios for health spending as % of GDP, 2009–2019. Supplemental Fig. 4. Four forecasted scenarios for health spending as % of GDP, for health care expendituresand long-term care expenditures. Supplemental Fig. 5. Four scenarios for spending varying the contribution of GDP per capita growth and residual growth. [file 12916_2025_3917_MOESM1_ESM.docx]

**Additional file 1**

Supplements to: Kinge et al. Forecasting total and cause-specific health expenditures for 116 health conditions in Norway, 2022-2050

**Part 1: Determinants of health and long-term care expenditures**

Historically, the growth in health expenditures in high-income nations has primarily been attributed to three key drivers: national income, technology, and demographic/epidemiological changes. Extensive research on the role of national income as a driver of health spending, suggests that higher national income increases the demand for better and more accessible health services, thereby escalating health care spending. Numerous studies have provided various estimates of this effect [1-4]. In addition to income, a proxy for “need for care” due to diseases or disabilities are often included. Traditionally, this need has been represented by age and proximity to death [1, 5].

Several studies highlight advancements in medical technology as a significant driver of health spending [1, 2, 5]. New technologies enable treatments for conditions previously untreatable, thereby increasing the relative cost (price) of treating each case as new, costly treatments become available. Additionally, technological advancements may reduce mortality rates and alter disease prevalence, leading to future unrelated medical costs during the additional life years gained [6]. For example, if new technology prevents deaths from ischemic heart disease, more people may live to older ages and require care for conditions like dementia. Thus, the effect of technology is intertwined with other drivers.

A final driver of spending is the ‘Baumols effect’. Given the labor-intensive nature of the health service sector (especially long-term care), it is often characterized as a low productivity sector. Over time, wages in low productivity sectors tend to rise in line with those in the broader economy, leading to an increasing ratio of spending to GDP in this sector [2, 7]. This Baumols effect is sometimes modeled separately, but it is also affected by GDP and is this sometimes modeled as a part of the effect of GDP on health spending [5].

**Part 2: More details about the forecasting methodology**

Given the evidence of the strong relationships between health expenditures and the drivers outlined in Part 1, these drivers form the basis of our model. First, our model incorporates the total population, age structure and prevalence of diseases directly. Second, we allow unit cost of care to develop with Gross Domestic Product (GDP). Following De la Maisonneuve and Oliveira Martins [5] assume that GDP directly impact on health spending via increased living standards, which can be considered a demand effect. In addition, we assume that increased GDP affect the supply side through the Baumols effect. I.e. with increasing GDP typically follows relative productivity gains in the rest of the economy and growth in wages. Wages in the health sector grow with the rest of the economy and thereby health spending (Baumols effect). Third, to account for the effect of technology, we employed the residual approach used by the Dybczak and Przywara [2]. This approach attributes changes not explained by population, ageing, disease prevalence, and GDP to medical technological progress. Practically, this involves methods to estimate the changes in health spending over time adjusted for the impact of epidemiological/demographic changes and income. Any residual growth is then attributed the technological changes.

Given that we have available forecasts of population, ageing, disease prevalence and GDP from other sources, we need to estimate how the spending varies with changes in GDP per capita and technology. To do this we used aggregate data for the years 1990-2019 for Norway, Sweden, and Denmark. Data for three countries were included in part of the model, to increase precision and make the model less vulnerable to random noise and/or policies. The model is developed to estimate how the cost of treating one case, change with GDP per capita and technology, adjusted for changes in the type and severity of diseases. Following the literature, we estimate the following models [1, 2, 8] :

1. ${lnHCE}_{c,t}=\alpha^{LTC}+\beta_{1}^{HCE}{lnGPD}_{c,t}+\beta_{2}^{HCE}{trend}_{t}+X_{c,t}^{'}\gamma^{HCE}+\gamma_{c}^{HCE}+\varepsilon_{c,t}^{HCE}$,
2. ${lnLTC}_{c,t}=\alpha^{LTC}+\beta_{1}^{LTC}{lnGPD}_{c,t}+\beta_{2}^{LTC}{trend}_{t}+X_{c,t}^{'}\gamma^{LTC}+\gamma_{c}^{LTC}+\varepsilon_{c,t}^{LTC}$

where lnHCE and lnLTC are the logarithms of health care and long-term care spending per prevalent case in country c, at time t, while income elasticity on lnGDP per capita is measured by β_1_. The impact of temporal growth, which primarily reflects the impact of technological advancements, on health expenditure is estimated by β_2_ deterministic time trend, which vary by type of care [2, 9]. $X_{c,t}^{'}$ are a vectors of control variables for the proportion of proportion with non-communicable diseases; proportion with infectious diseases; deaths per prevalent case and cases per person. The vectors of control variables are included to adjust for the type and severity of diseases. The primary estimation method is a Mixed Linear Regression fitted using reduced maximum likelihood allowing for country random intercepts ($\gamma_{c})$ [10]. However, the specification of such regressions will potentially have substantial effects on the forecasts and have differed substantially in prior literature [2, 9, 11]. Hence, we show the effects of different specifications in Supplemental Table 2. With and without various covariates and also a model were $\gamma_{c}$ are included as country fixed effects.

*Non-stationarity and cointegration*

Prior studies have found that time-series of national health care spending per case and GDP per capita are non-stationary, which might lead to spurious relationships between them [2, 4]. However, this potential issue disappears when the variables are cointegrated, which means that there is a long run relationship between them [3, 12].We perform the Kao test of no cointegration and can reject this for both health and long term care spending (p-values <0.05).

*Imputing data*

Data on long-term care spending was not available for all countries from 1990-2019. We imputed long-term care spending based on total health care spending and trends in the associations between LTC and HCE spending. We bootstrapped this imputation in combination with eq. (1) and (2) to adjust the standard errors. We also supplemented the OECD data with data from Statistics Sweden.

**Part 3: The cause list**

The cause list for this project was based on the Disease Expenditure (DEX) project at the Institute for Health Metrics and Evaluation [13]. Their cause list is in turn based on the Global Burden of Disease Study 2017. GBD 2017 classified causes of health loss at five different levels of disaggregation. The DEX project extracted the Level III classification from GBD 2017. This resulted in a list of 140 causes based on GBD alone. An additional 14 causes were added to the DEX project to account for 4 risk factors and 10 health conditions associated with spending, even if not accounted for by the burden focused GBD project: hypertension, hyperlipidemia, obesity, and tobacco cessation. In addition to these, 10 causes were added that were not associated with health loss but were associated with health spending. Examples of these 10 additional causes were routine health check-ups and uncomplicated labor and delivery, heart failure, septicemia, and renal failure. Spending on these “causes” was tracked because they represent large portions of health spending and are of political interest. However, a modified version of this cause list has been used, in which these extra causes has been collapsed into three categories. Furthermore, the study by Kinge, Dieleman [14] presented only four categories for injuries, which has been collapsed into three for this study. A detailed list and map of all causes is in Supplemental Table 2.

**Supplemental Table 1: Data sources used in this study**

| **Data source** | **Variables** | **Years** | **Used in equations** |
| --- | --- | --- | --- |
| The Norwegian disease specific health expenditure study [14] | Health expenditures by health conditions, age and sex | 2019 | 3,4 |
| OECD Health expenditure and financing [15] | National health expenditures | 1990-2021 | 1,2,3 |
| OECD Demography and population [16] | Proportion above 65 | 1990-2021 | 1,2 |
| IHME Gross Domestic Product Per Capita 1960-2050 [17] * | GDP per capita and uncertainty intervals | 2022-2050 | 1,2,3 |
| IHME prevalence by disease [18] | Prevalence | 1990-2019 | 1, 2 |
| OECD national accounts [19] Economic Outlook 109 database [20] | GDP and GDP deflator | 1990-2022 | 1, 2, 3 |
| Statistics Sweden [21] | National health expenditures | 2001-2019 | 1, 2 |
| Statistics Norway [22] | National health expenditures | 2020-2023 | 1,2, 3 |
| Vollset, Ababneh [23] | Population by age and prevalence | 2020-2050 | 3,4 |

* Used version Y2023M01D13 of this data

**Supplemental Table 2: Aggregated to disaggregated reporting level**

| **Aggregate conditions (Level 2)** | **Disaggregated conditions (Level 3)** |
| --- | --- |
| Communicable, maternal, neonatal, and nutritional diseases | Tuberculosis |
|  | HIV/AIDS |
|  | Diarrheal diseases |
|  | Tetanus |
|  | Measles |
|  | Varicella |
|  | Intestinal infectious diseases |
|  | Lower respiratory tract infections |
|  | Upper respiratory tract infections |
|  | Otitis media |
|  | Meningitis |
|  | Encephalitis |
|  | Diphtheria |
|  | Whooping cough |
|  | Neglected tropical diseases and malaria |
|  | Maternal hemorrhage |
|  | Maternal sepsis and other pregnancy related infection |
|  | Hypertensive disorders of pregnancy |
|  | Obstructed labor |
|  | Complications of abortion |
|  | Pre-existing medical condition complicating pregnancy or childbirth |
|  | Other maternal disorders |
|  | Preterm birth complications |
|  | Neonatal encephalopathy (birth asphyxia and birth trauma) |
|  | Sepsis and other infectious disorders of the newborn baby |
|  | Hemolytic disease in fetus and newborn and other neonatal jaundice |
|  | Other neonatal disorders |
|  | Protein-energy malnutrition |
|  | Iodine deficiency |
|  | Vitamin A deficiency |
|  | Iron-deficiency anemia |
|  | Other nutritional deficiencies |
|  | Sexually transmitted diseases excluding HIV |
|  | Hepatitis |
|  | Leprosy |
|  | Other infectious diseases |
| Neoplasms | Esophageal cancer |
|  | Colon and rectum cancers |
|  | Mouth cancer |
|  | Nasopharynx cancer |
|  | Other pharynx cancer |
|  | Gallbladder and biliary tract cancer |
|  | Pancreatic cancer |
|  | Malignant skin melanoma |
|  | Non-melanoma skin cancer |
|  | Ovarian cancer |
|  | Testicular cancer |
|  | Stomach cancer |
|  | Kidney cancer |
|  | Bladder cancer |
|  | Brain and nervous system cancers |
|  | Thyroid cancer |
|  | Hodgkin lymphoma |
|  | Non-Hodgkin lymphoma |
|  | Multiple myeloma |
|  | Leukemia |
|  | Other neoplasms |
|  | Liver cancer |
|  | Larynx cancer |
|  | Trachea, bronchus, and lung cancers |
|  | Breast cancer |
|  | Cervical cancer |
|  | Uterine cancer |
|  | Prostate cancer |
| Other non-communicable diseases | Congenital anomalies |
|  | Skin and subcutaneous diseases |
|  | Sense organ diseases |
|  | Oral disorders |
| Cardiovascular diseases | Rheumatic heart disease |
|  | Other cardiovascular and circulatory diseases |
|  | Ischemic heart disease |
|  | Cerebrovascular disease |
|  | Hypertensive heart disease |
|  | Cardiomyopathy and myocarditis |
|  | Atrial fibrillation and flutter |
|  | Aortic aneurysm |
|  | Peripheral vascular disease |
|  | Endocarditis |
| Chronic respiratory diseases | Chronic obstructive pulmonary disease |
|  | Pneumoconiosis |
|  | Asthma |
|  | Interstitial lung disease and pulmonary sarcoidosis |
|  | Other chronic respiratory diseases |
| Cirrhosis of the liver | Cirrhosis of the liver |
| Digestive diseases | Peptic ulcer disease |
|  | Other digestive diseases |
|  | Appendicitis |
|  | Paralytic ileus and intestinal obstruction |
|  | Inguinal or femoral hernia |
|  | Inflammatory bowel disease |
|  | Vascular intestinal disorders |
|  | Gallbladder and biliary diseases |
|  | Pancreatitis |
|  | Gastritis and duodenitis |
| Neurological disorders | Alzheimer disease and other dementias |
|  | Parkinson’s disease |
|  | Epilepsy |
|  | Multiple sclerosis |
|  | Migraine |
|  | Tension-type headache |
|  | Other neurological disorders |
| Mental and substance use disorders | Schizophrenia |
|  | Conduct disorder |
|  | Idiopathic intellectual disability |
|  | Other mental and behavioral disorders |
|  | Alcohol use disorders |
|  | Drug use disorders |
|  | Depressive disorders |
|  | Bipolar disorder |
|  | Anxiety disorders |
|  | Eating disorders |
|  | Autistic spectrum disorders |
|  | Attention- deficit/hyperactivity disorder |
| Diabetes, urogenital, blood, and endocrine diseases | Diabetes mellitus |
|  | Acute glomerulonephritis |
|  | Chronic kidney diseases |
|  | Urinary diseases and male infertility |
|  | Gynecological diseases |
|  | Hemoglobinopathies and hemolytic anemias |
|  | Endocrine, metabolic, blood, and immune disorders |
| Musculoskeletal disorders | Rheumatoid arthritis |
|  | Low back and neck pain |
|  | Gout |
|  | Osteoarthritis |
|  | Other musculoskeletal disorders |
| Injuries | Transport injuries |
|  | Unintentional injuries |
|  | Self-harm and interpersonal violence |
| Well care & pregnancy related care | Well care & pregnancy related care |
| Treatment of risk factors (tobacco, obesity, hypertension & hyperlipidemia) | Treatment of risk factors (tobacco, obesity, hypertension & hyperlipidemia) |
| Impairments (Heart failure, septicemia, renal failure) | Impairments (Heart failure, septicemia, renal failure) |

**Supplemental Table 3: Regression results, coefficients and standard errors**

|  | Health care spending per case | | | | | | | | | | |  | LTC spending per case | | | | | | | | | | |
| --- | --- | --- | --- | --- | --- | --- | --- | --- | --- | --- | --- | --- | --- | --- | --- | --- | --- | --- | --- | --- | --- | --- | --- |
| Country RE/FE | RE | |  | RE | |  | RE | |  | FE | |  | RE | |  | RE | |  | RE | |  | FE | |
|  | Coef. | Std.err. |  | Coef. | Std.err. |  | Coef. | Std.err. |  | Coef. | Std.err. |  | Coef. | Std.err. |  | Coef. | Std.err. |  | Coef. | Std.err. |  | Coef. | Std.err. |
| GDP per capita | 1.398916 | .062565 |  | .533328 | .0964395 |  | .5539365 | .0875918 |  | .4736501 | .4736501 |  | 2.526309 | .1054698 |  | 1.775804 | .2286775 |  | 1.770164 | 0.1829151 |  | 1.893439 | .236736 |
| Temporal trend |  |  |  | .0151135 | .0015184 |  | .0131623 | .0025076 |  | .0158501 | .0036352 |  |  |  |  | .0126113 | .003588 |  | .0077314 | .0052935 |  | .0032863 | .0074173 |
| Causes proportion |  |  |  |  |  |  |  |  |  |  |  |  |  |  |  |  |  |  |  |  |  |  |  |
| Injuries | - | - |  | - | - |  | - | - |  | - | - |  | - | - |  | - | - |  | - | - |  | - | - |
| Infectious diseases |  |  |  |  |  |  | -.2584324 | .1029464 |  | -.2256328 | .1180738 |  |  |  |  |  |  |  | 1.006899 | .2124026 |  | .8732769 | .2409152 |
| NCDs |  |  |  |  |  |  | -.0864364 | .0400516 |  | -.0524371 | .0479907 |  |  |  |  |  |  |  | .4064585 | .0829336 |  | .3806597 | .0979191 |
| Deaths per case |  |  |  |  |  |  | -29.47972 | 23.78361 |  | -7.258984 | 31.03395 |  |  |  |  |  |  |  | 15.6609 | 49.5951 |  | -13.9872 | 63.32097 |
| Cases per person |  |  |  |  |  |  | -1.403965 | 1.974274 |  | -2.351955 | 2.131059 |  |  |  |  |  |  |  | 4.412748 | 4.072536 |  | 3.126801 | 4.348166 |
| Constant | -7.354511 | .7275769 |  | 2.372139 | 1.090402 |  | 12.31507 | 4.313084 |  | 10.79946 | 4.602678 |  | -21.40452 | 1.246014 |  | -12.96343 | 2.586347 |  | -56.32073 | 8.830456 |  | -53.36692 | 9.391201 |

FE: country fixed effects. RE: country random effects

**Supplemental Table 4: Forecast performance measures**

| Measure | Total health spending | | |  | Health spending as % of GDP | | |
| --- | --- | --- | --- | --- | --- | --- | --- |
|  | Reference | Cost pressure | Cost containment |  | Reference | Cost pressure | Cost containment |
| Root Mean Squared Error (RMSE) | 14208449536 | 13698438144 | 26272661504 |  | .43033275 | .41744334 | .75783491 |
| Mean Absolute Error (MAE) | 12964898406 | 12383848038 | 21552503194 |  | .39137602 | .375179 | .62853212 |
| Mean Absolute Percentage Error (MAPE) | 4.35% | 4.21% | 6.58% |  | 4.35% | 4.21% | 6.58% |
| R^2^ | 0.922 | .920 | .923 |  | .804 | .801 | .776 |

**Supplemental Figure 1: Forecasted growth in GDP per capita, with uncertainty intervals.**

Source IHME [17].

**Supplemental Figure 2: Historic and four forecasted scenarios for health spending, 2009-2019.**


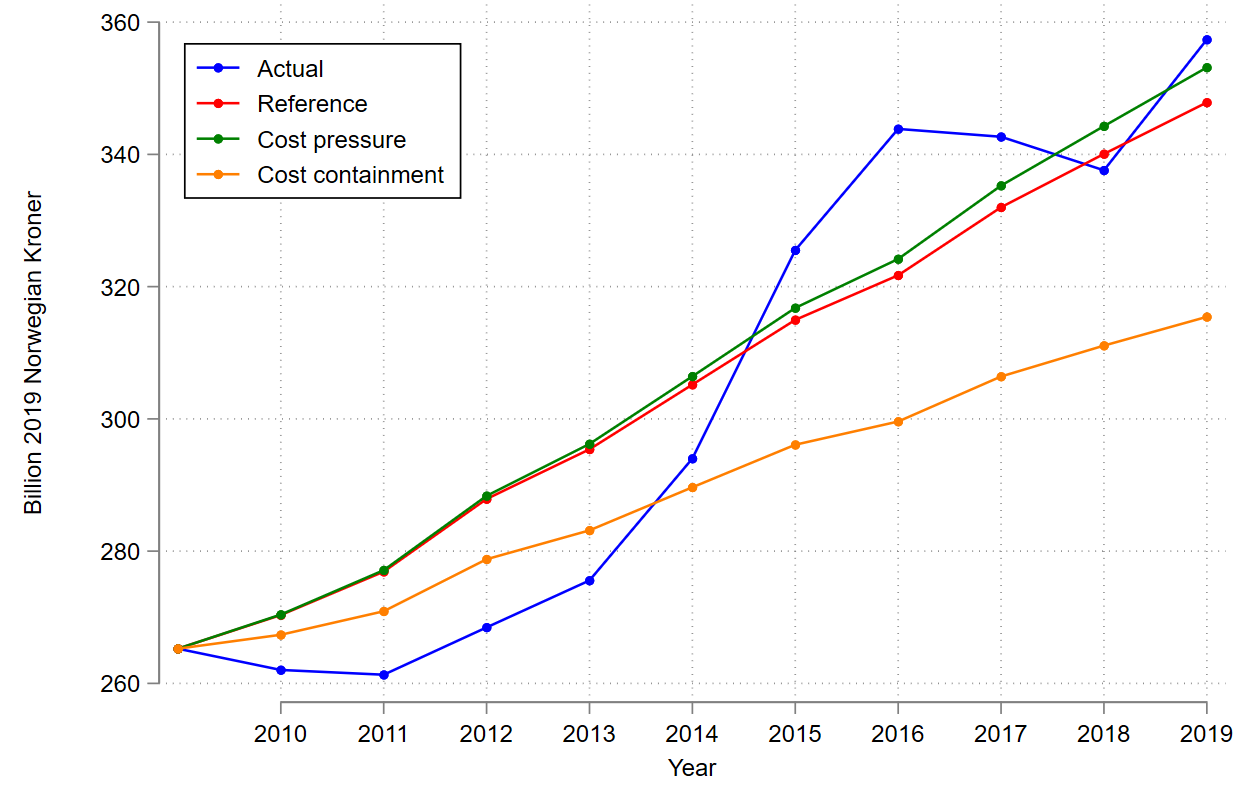


**Supplemental Figure 3: Historic and four forecasted scenarios for health spending as % of GDP, 2009-2019.**


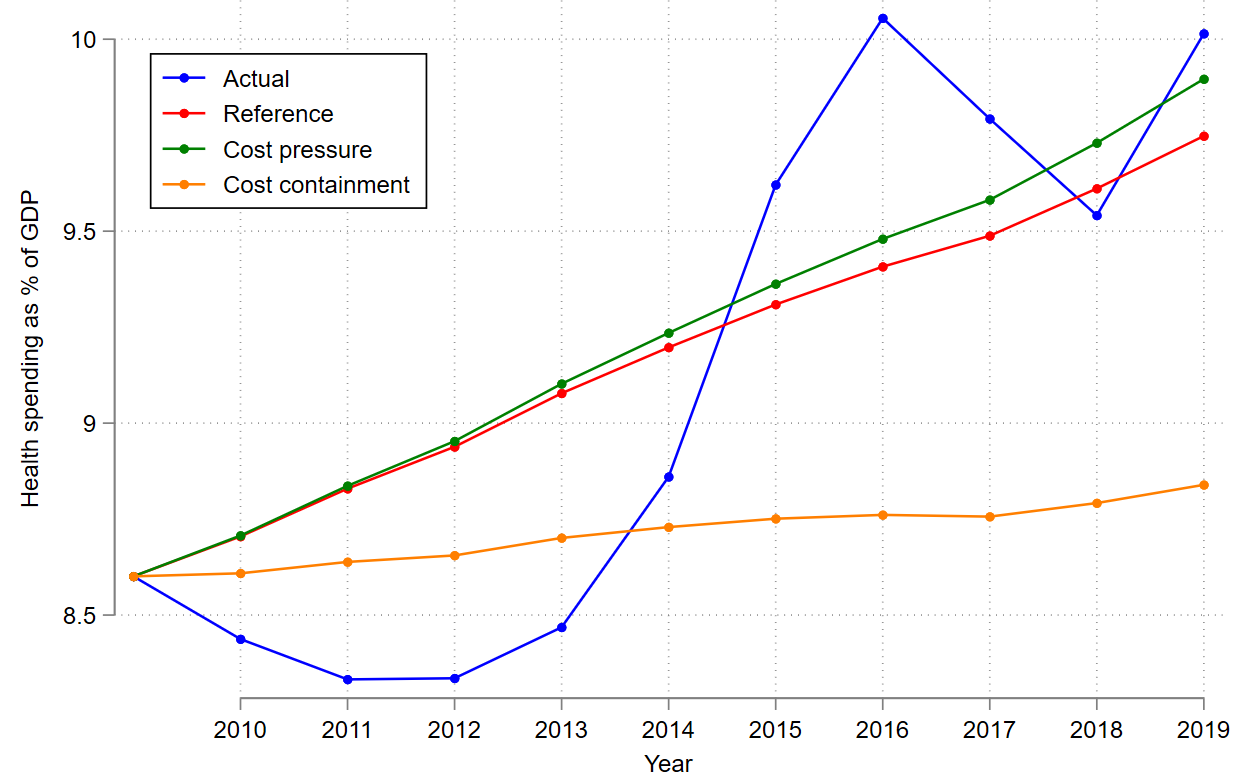


**Supplemental Figure 4: Four forecasted scenarios for health spending as % of GDP, for health care expenditures (A) and long-term care expenditures (B)**

A B


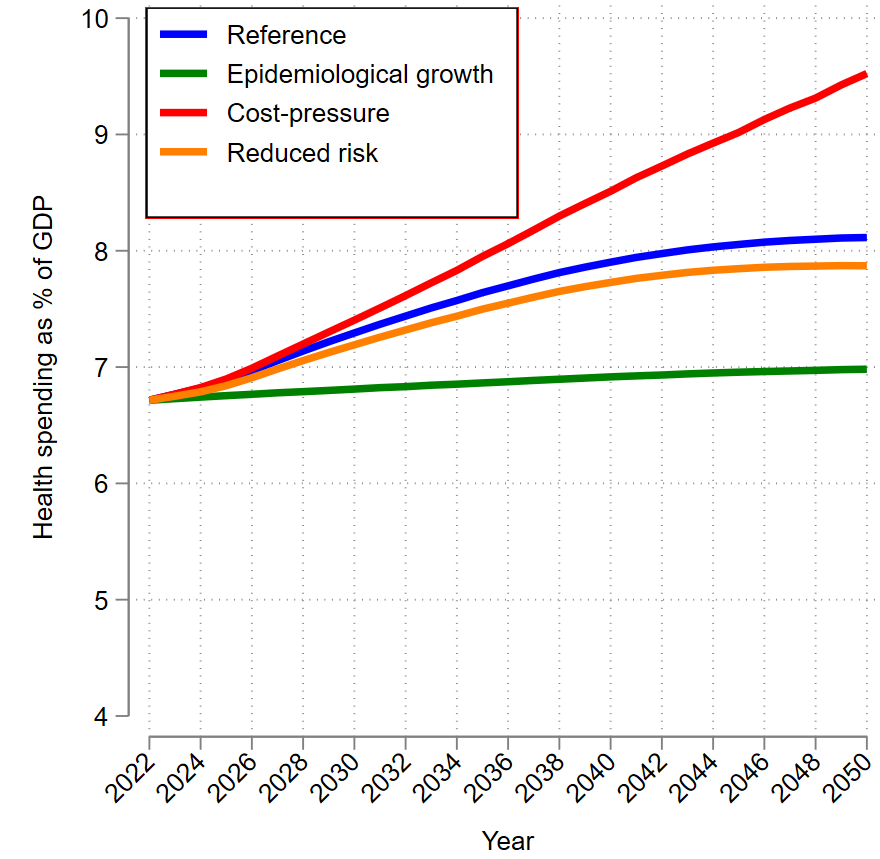

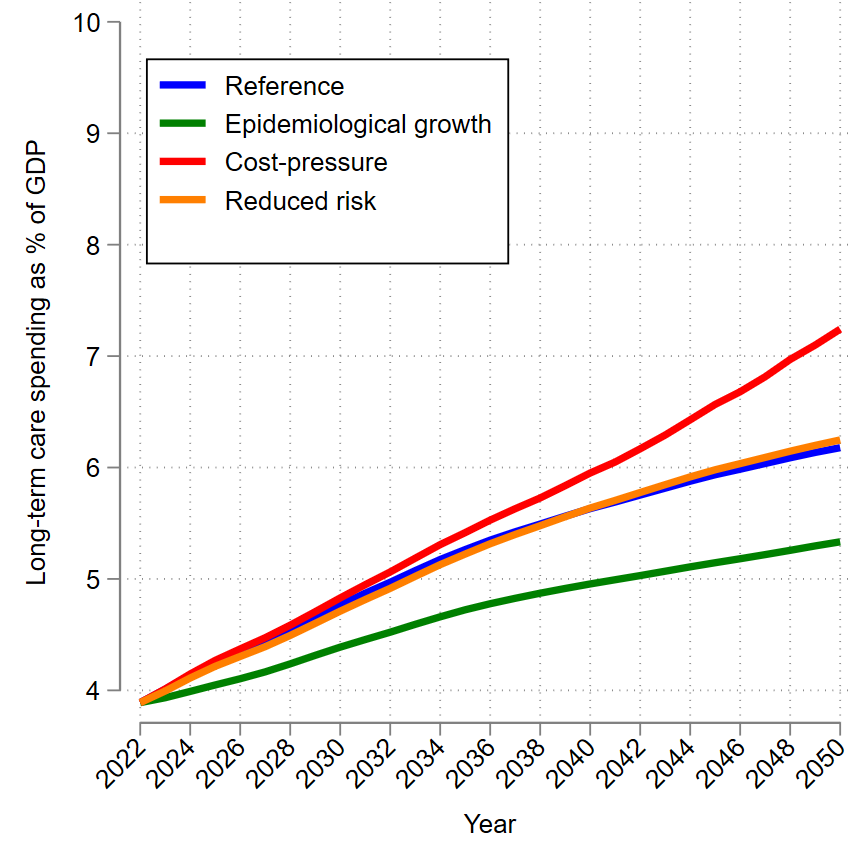


**Supplemental Figure 5: Four scenarios for spending varying the contribution of GDP per capita growth and residual growth.**

Total health spending (A) Curative care spending (B)

Long-term care spending (C)

**References:**

1. Lorenzoni, L., et al., *Health Spending Projections to 2030: New results based on a revised OECD methodology.* 2019.

2. Dybczak, K. and B. Przywara, *The role of technology in health care expenditure in the EU*. 2010, Directorate General Economic and Financial Affairs, European Commission.

3. Gerdtham, U.-G. and M. Löthgren, *On stationarity and cointegration of international health expenditure and GDP.* Journal of Health Economics, 2000. **19**(4): p. 461-475.

4. Feng, Y., et al., *What Determines the Health Care Expenditure of High Income Countries? A Dynamic Estimation.* Applied Economics and Finance, 2017.

5. De la Maisonneuve, C. and J. Oliveira Martins, *A projection method for public health and long-term care expenditures.* 2013.

6. Perry-Duxbury, M., et al., *Cured today, ill tomorrow: a method for including future unrelated medical costs in economic evaluation in England and Wales.* Value in Health, 2020. **23**(8): p. 1027-1033.

7. Baumol, W.J., *Health care, education and the cost disease: A looming crisis for public choice*, in *The next twenty-five years of public choice*. 1993, Springer. p. 17-28.

8. Costa-Font, J. and C. Vilaplana-Prieto, *‘Investing’in care for old age? An examination of long-term care expenditure dynamics and its spillovers.* Empirical Economics, 2023. **64**(1): p. 1-30.

9. Okunade, A.A. and V.N. Murthy, *Technology as a ‘major driver’of health care costs: a cointegration analysis of the Newhouse conjecture.* Journal of health economics, 2002. **21**(1): p. 147-159.

10. Thompson Jr, W., *The problem of negative estimates of variance components.* The Annals of Mathematical Statistics, 1962: p. 273-289.

11. Heffler, S., Caldis, TG., Smith, SD., Cuckler, GA., *The Long-Term Projection Assumptions for Medicare and Aggregate National Health Expenditures*, D.O.H.A.H. SERVICES, Editor., Centers for Medicare & Medicaid Services: Baltimore, MD.

12. Wooldridge, J.M., *Introductory Econometrics: A Modern Approach 3rd ed*. 1996.

13. Dieleman, J.L., et al., *US health care spending by payer and health condition, 1996-2016.* Jama, 2020. **323**(9): p. 863-884.

14. Kinge, J.M., et al., *Disease-specific health spending by age, sex, and type of care in Norway: a national health registry study.* BMC medicine, 2023. **21**(1): p. 201.

15. OECD, *OECD Health Statistics—Health Expenditure and Financing*, OECD, Editor. 2023: Paris.

16. OECD, *Demography and poplation*, OECD, Editor. 2023: Paris.

17. Global Burden of Disease Collaborative Network, *Gross Domestic Product Per Capita 1960-2050*, I.f.H.M.a.E. (IHME), Editor. 2023: Seattle, United States of America.

18. Institute for Health Metrics and Evaluation (IHME), *GBD Results*, U.o.W. HME, Editor. 2020: Seattle, WA.

19. OECD, *National accounts*, OECD, Editor. 2023: Paris.

20. OECD, *Economic Outlook 109 database*, OECD, Editor. 2021: Paris.

21. Statistics Sweden, *System of Health Accounts (SHA)*, S. Sweden, Editor. 2023: Örebro.

22. Statistics Norway, *Health accounts*, Statistics Norway, Editor. 2023: Oslo.

23. Vollset, S.E., et al., *Burden of disease scenarios for 204 countries and territories, 2022-2050: a forecasting analsysi for the Global Burden of Disease Study.* The Lancet, 2024. **403**(10440): p. 2204 - 2256.
